# Adenosine-A2A Receptor Pathway in Cancer Immunotherapy

**DOI:** 10.3389/fimmu.2022.837230

**Published:** 2022-03-21

**Authors:** Changfa Sun, Bochu Wang, Shilei Hao

**Affiliations:** Key Laboratory of Biorheological Science and Technology, Ministry of Education, College of Bioengineering, Chongqing University, Chongqing, China

**Keywords:** adenosine, A2AR, antagonist, immunosuppression, tumor microenvironment

## Abstract

A2A receptors (A2AR), a typical GPCR with a high affinity for adenosine, was expressed in many immune cells, such as regulatory T cells, cytotoxic T cells, macrophages, etc. Adenosine binding to the A2AR receptor activates the typical G protein and triggers the cAMP/PKA/CREB pathway. The adenosine-A2AR pathway plays an important role in protecting normal organs and tissues from the autoimmune response of immune cells. However, many solid tumors hijack the adenosine-A2AR pathway by promoting adenosine accumulation. The activation of the A2AR pathway inhibited the immune response of immune cells and then promotes the immune escape of tumor cells in the tumor microenvironment. Recently, both animal experiments and clinical trials indicated that blocking the adenosine pathway can inhibit the progression of a variety of solid tumors. In addition, it is encouraging that A2AR blockade combined with CAR T cells therapy showed better anti-tumor efficacy. Therefore, this review will discuss the role of the adenosine-A2AR pathway in the tumor microenvironment and summarize recent advances of A2AR-cancer related studies.

## Introduction

Targeting the adenosine-A2AR pathway is one of the few novel approaches that holds great promise for saving all patients with tumors that are refractory to other therapies, including PD1 blockade, and recent clinical evidence suggests that this approach is effective (1), although much work remains to be done to improve it. Adenosine is an important intermediate for the synthesis of adenosine triphosphate (ATP), adenine, and adenylate (2).In addition, extracellular adenosine also acts as a signal molecule to activate adenosine receptors and plays a more extensive physiological role (3). Adenosine receptor, a GPCR, has been found in four categories: A1R, A2AR, and A3R with high affinity for adenosine, and A2BR with low affinity for adenosine (4–6). Among them, the A2AR is widely distributed on the surface of most immune cells (7–9). The discovery and development of the Hypoxia-A2-adenosinergic Pathway in cancer immunotherapy goes through three phases.

Phase I, the discovery and demonstration of the principle of adenosine -A2AR pathway mediated immunosuppression. Extracellular adenosine binds to A2AR on the surface of immune cells and activates the downstream cAMP pathway, which could lead to inhibition of T cells activation and expansion (10). The adenosine-A2AR pathway is critical and non-redundant in controlling inflammatory damage to overactive anti-pathogen immune cells (11), yet the axis also reduces damage to cancer tissue by anti-tumor killer cells. Studies have shown that the solid tumor microenvironment often presents with transient or chronic hypoxia (12, 13), which facilitates the accumulation of extracellular adenosine (14). Ohta’s team first propose to target the hypoxia-adenosine-A2AR pathway as a cancer immunotherapy strategy and demonstrated that pharmacological or genetic elimination of the extracellular adenosine-A2AR-cAMP axis significantly improves T cell rejection of tumors in an *in vivo* model of tumor immunotherapy (15).

Phase II, the confirmations, and extensions to clinical trials. CD39 (also known as ENTPD1) being upstream of CD73 in the CD39/CD73 tandem of ectoenzyme, is the dominant ectoenzyme that controls extracellular nucleotide concentrations, which is expressed on the surface of T regulatory cells and generates adenosine to T effector cells with A2AR expression (16–18). Robson’s team demonstrated for the first time that inhibition of extracellular adenosine-mediated signal transduction in CD39-deficient mice affected angiogenesis and tumor growth (19). In addition, Cd39 deletion of bone marrow-derived cells can enhance NK cell-mediated antitumor immunity and inhibit liver metastasis of melanoma (20). Zhang Bin’s team confirmed that CD73-generated adenosine prevents tumor destruction by inhibiting antitumor immunity and CD73 expression in tumor cells negatively regulates T cell response, and knockdown of CD73 expression can prolong the survival of tumor-bearing mice and enhance adoptive T-cell therapy (21). In the same year team of Mark Smyth demonstrated that anti-CD73 antibody therapy inhibits breast tumor growth and metastasis in a mouse model (22). However, it’s worth noting that the blockade of CD73 therapy not only requires adaptive immunity (15, 23) but also requires A2A adenosine receptors on immune cells (22, 24). In addition, anti-hypoxic oxygenation agents or hyperoxic breathing can also act as an “Anti-A2-adenosinergic drug” by inhibiting the expression of HIF-1α, which is upstream of CD39 and CD73 (25–27).

Phase III, the current clinical transformation. The following are some of the methods for blocking or reducing the immunosuppression caused by the Hypoxia/HIF-1α -A2-Adenosinergic pathway. ①Blocking Hypoxia agents, ②Blocking CD39 agents ③Blocking CD73 agents ④A2AR/A2BR antagonist ⑤Block PKA agents (28). Preliminary clinical results have reported that some agents [including anti-CD73 antibody, BMS-986179 (29) and CPI-006 (30); A2AR antagonist NIR178 (PBF-509) (31), inupadenant (EOS-850) (32) and Ciforadenant (CPI-444) (1, 33); A2AR/A2BR dual antagonist, AB928 (34, 35)] show good human tolerability and encouraging cancer treatment effects.

## 1 Hypoxia and Extracellular Adenosine Accumulation in Cancer

Multiple cell types release adenine nucleotides in the form of ATP, ADP, and AMP when exposed to hypoxia. In addition, intratumoral hypoxia can result in HIF-1α overexpression (36), which promotes the transcription of genes [including CD39 and CD73 which represent the major source of extracellular adenosine in the tumor microenvironment (25)] implicated in important aspects of cancer biology (37).

Here, we summarize the metabolic patterns of extracellular adenosine. There are three ways to produce extracellular adenosine: ① ATP generates ADP under the catalysis of CD39, and ADP continues to generate amp under the catalysis of CD39; AMP generates adenosine under the catalysis of CD73, which is also the most important way of adenosine production. ② nicotinamide adenine dinucleotide (NAD+) generates ADPR through CD38, ADPR generates amp under the catalysis of CD203a, and amp generates adenosine under the action of CD73 ③ S-adenosylhomocysteine (SAH) generates adenosine under S-adenosylhomocysteine hydrolase (SAHH). This enzymatic reaction process is reversible. The metabolism and utilization of adenosine mainly include: ① adenosine is transformed into AMP through adenosine kinase (AK). ② Adenosine is converted to inosine by adenosine deaminase (ADA).

**Figure 1:**
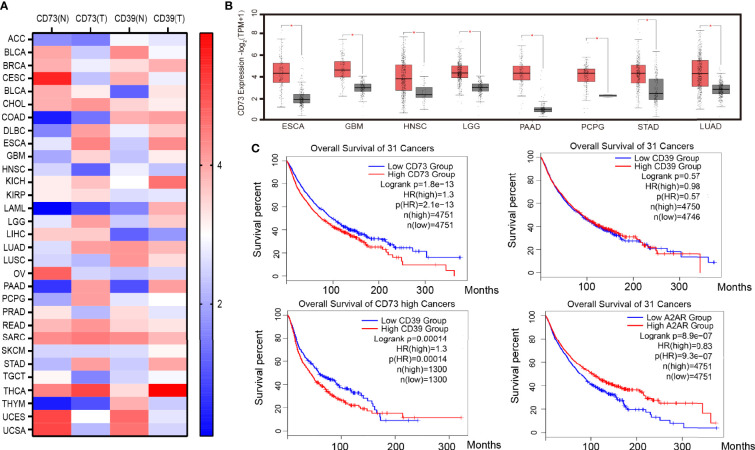
CD39, CD73, and A2AR expression and survival in cancer. **(A)** Heat map of CD39 and CD73 genes expressed in 31 kinds of tumor tissue and corresponding normal tissue. **(B)** Cancers with significantly high expression of the CD73 gene. **(C)** Survival analysis of cancer patients with high or low CD39, CD73 and A2AR genes expression. Data from the TCGA and GTEx studies on RNA sequencing expression in 9,736 cancers and 8,587 normal samples. ACC (Adrenocortical carcinoma), BLCA (Bladder Urothelial Carcinoma), BRCA (Breast invasive carcinoma), CESC (Cervical squamous cell carcinoma and endocervical adenocarcinoma), CHOL (Cholangio carcinoma), COAD (Colon adenocarcinoma), DLBC (Lymphoid Neoplasm Diffuse Large B-cell Lymphoma), ESCA (Esophageal carcinoma), GBM (Glioblastoma multiforme), HNSC (Head and Neck squamous cell carcinoma), KICH(Kidney Chromophobe), KIRC (Kidney renal clear cell carcinoma), KIRP (Kidney renal papillary cell carcinoma), LAML (Acute Myeloid Leukemia), LGG (Brain Lower Grade Glioma), LIHC (Liver hepatocellular carcinoma), LUAD (Lung adenocarcinoma), LUSC (Lung squamous cell carcinoma), MESO (Mesothelioma), OV (Ovarian serous cystadenocarcinoma), PAAD (Pancreatic adenocarcinoma), PCPG (Pheochromocytoma and Paraganglioma), PRAD (Prostate adenocarcinoma), READ (Rectum adenocarcinoma), SARC (Sarcoma), SKCM (Skin Cutaneous Melanoma), STAD (Stomach adenocarcinoma), TGCT (Testicular Germ Cell Tumors), THCA (Thyroid carcinoma), THYM (Thymoma), UCEC (Uterine Corpus Endometrial Carcinoma), UCS (Uterine Carcinosarcoma).

Under the environmental induction of hypoxia and high H +, many tumor cells, such as GBM, LGG, PAAD, and STAD (**Figures 1A, B**), upregulate the expression of CD39 and CD73. The increased expression of CD73 and CD39 on the tumor surface will lead to the production of adenosine. In addition, hypoxia can also reduce the expression of adenosine kinase and inhibit the transformation of adenosine (38). A high concentration of adenosine further reduced the expression of ADA/CD26 complex, formed positive feedback, and promoted adenosine accumulation (39, 40) Moreover, a large number of experiments have proved that the concentration of adenosine in the tumor microenvironment is much higher than that in normal tissues (41). The accumulation of adenosine was thought to be a major cause of tumor immunosuppression and escape. The median survival time of patients with high CD73 expression in 31 cancers (the median overall survival time is 74.3 months) is significantly lower than that of patients with low CD73 expression (the median overall survival time is 98.3 months), according to survival analysis of TCGA data. Patients with low CD39 expression did not have a better survival prognosis than patients with high CD39 expression, according to the overall survival analysis of CD39 in 31 cancers. However, when the median overall survival time of CD39-high patients was compared to that of CD39-low patients in eight cancers with high CD73 expression, it was discovered that the median overall survival time of CD39-high patients was significantly shorter than that of CD39-low patients (the median overall survival 42.4 months *VS* 58.6 months). As a result, we believe that CD73, rather than CD39, was a more critical component in adenosine buildup and tumor immunosuppression. Although CD39 is a crucial step in the synthesis of adenosine, its role was redundant, and the CD38-CD203a-CD73 pathway might partially replace CD39’s activity (42).

## 2 A2AR-Adenosine Pathway in Cancer

### 2.1 Adenosine- A2AR Signaling in Tumor Cells

CD73 promotes the accumulation of adenosine, which activates the A2AR signal pathway in tumor cells, and A2AR signal activation leads to the activation of Rap1, which recruits P110 to the plasma membrane and triggers PIP3 production. After that, the PIK3/AKT signaling pathway is turned on (43). The activation of the PIK3/AKT signal promotes tumor cell EMT (Epithelial-Mesenchymal Transition) and anti-apoptosis, which promote tumor cell growth and metastasis (44). Furthermore, a TCGA survival analysis revealed that tumor patients with high A2AR expression had a worse prognosis (median overall survival was 77.8 months versus 104.6 months) (**Figure 1C**), implying that tumor cells’ adenosine-A2AR pathway promotes tumor progression

### 2.2 Adenosine- A2AR Signaling in Immune Cells

**Figure 2:**
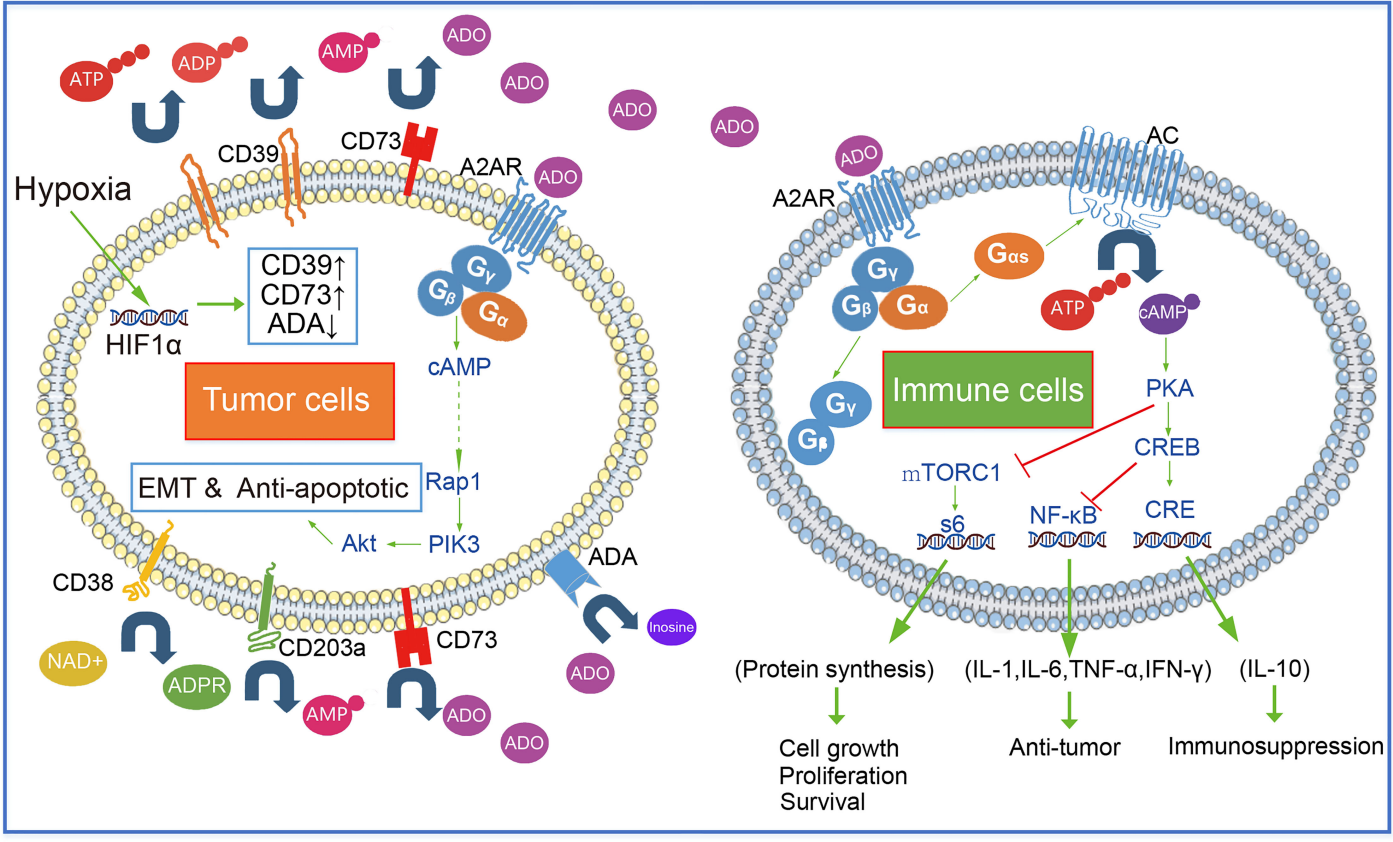
Extracellular adenosine- A2AR signal pathway in tumor and immune cells.

Immune cells recognize tumor cell surface antigens and then initiate cellular immunity and kill tumor cells by secreting anti-tumor-related cytokines (INF-γ, TNF-α, and IL-6, etc.) and cell phagocytosis. Most immune cells express A2AR on the surface. As GPCR, A2AR on the surface of the immune cell together with intracellular G_α_ (mainly G_αs_ subunit) and G_β_-G_γ_ form a complex. G_αs_ dissociated with G_β_-G_γ_ after adenosine binding to A2AR. Dissociated Gαs activates AC enzymes (such as ADCY1 and ADCY9), AC decomposes intracellular ATP to produce diphosphate and cAMP. PKA is activated by cAMP, an intracellular second messenger. Under the action of PKA, CREB gets phosphorylated. Cre (cAMP response element) is bound by phosphorylated CREB and other proteins such as the CBP-p300 complex, and then IL-10, Foxp3, and other factors can begin to be expressed. Furthermore, suppression of phosphorylated CREB, can trigger the expression of cytokines such as TNF-α, IL-1β, IL-6, and NOS2 (**Figure 2**).

#### 2.2.1 T Cells Respond to the Adenosine - A2AR Signaling

Cellular immunity mediated by T-lymphocytic subsets such as CD4+ T and CTL is an important way for the body to eliminate the threat of tumor cells. Antigen activation of TCR on CD4+ T cells surface resulted in up-regulation of INF-γ, CD25, and CD69 expression (45), Adenosine inhibits TCR and IL-2 receptor triggered signal transduction by activating (46). In addition, *in vitro* experiments showed that the gene dose effect affected the inhibition degree. The maximum inhibition degree of A2AR-/+ CD4+ T lymphocytes by A2AR agonist was 50 percent, and the maximum inhibition degree of A2AR-/- CD4+ T lymphocytes was 100 percent (45). Naïve T cell is called Th0, it could develop into various CD4+ T cell subsets in response to various cytokines. CD4+ T cells can be classified into THL, Th2, Th17, Treg, Th9, and Th22 subsets based on the secreted cytokine spectrum. Among them, Th1 (secreting cytokines such as INF-γ, IL-2, TNF, and IL12) mainly mediates the cellular immune response. It is worth noting that INF-γ and IL-2 can not only promote the development and proliferation of Th1 but also inhibit the differentiation of Th0 into Th2 and Th17 (47). Activation of the adenosine -A2AR pathway leads to the reduction of Th1 induced by decreasing number of cytokines like INF-γ and IL-2. Adenosine and A2AR agonists(For example ATL146e, ATL370, and ATL1223)have been shown to enhance the TGFβ-induced generation of FoxP3+ Tregs (48). In addition to protecting the host from pathogen invasion, the human immune system is responsible for inhibiting the immune response to self-antigens and preventing an excessive immune response from causing damage to the host. And this activity is primarily mediated by regulatory T cells (Tregs) that specialize in immunosuppression (49, 50). However, the increase of CD4+ Foxp3+ Treg inhibited the antitumor effect (51). Blocking A2AR *in vivo* using the antagonist SCH58261 inhibited tumor growth, induced the decrease of CD4+ Foxp3+ Treg cells, and enhanced the antitumor response of T cells (52).

The main way of adenosine immunosuppression is to block the cytotoxic function of CD8+ T cells through the A2AR signal. This promotes the immune escape of tumor cells. In B16F10 and SM1WT1 tumor models of A2AR deficient mice, the infiltration of CD8+ T cells into tumors was enhanced, which inhibited the growth and metastasis of tumors (53). In an HNSCC mouse tumor model, it was found that blocking A2AR with SCH58261, a small molecule inhibitor could increase the number of tumor-infiltrating CD8+ T cells, at the same time, the function of CD8+ T cells was enhanced (INF-γ and TNF-α expression increased) (52). By analyzing adenosine-mediated the immunosuppression on CD8+ T cells at different differentiation stages (T central memory-T_CM_, T effector memory-T_EM_, T terminally differentiated-T_EMRA_), it was found that the expression level of A2AR in CD8+ T cells determined the sensitivity to adenosine, T_CM_ was more sensitive to adenosine, and its cytokine secretion was more inhibited than T_EM_ and T_EMRA_ subgroups (54). However, the inhibition degree of adenosine on T cell’s chemotaxis did not depend on the expression level of A2AR. The increased sensitivity of CD8+ T cells to adenosine was associated with the decrease of KCa3.1 potassium channel activity, but not with adenosine receptor expression or signal transduction. KCa3.1 channel agonist treatment can restore the migration of CD8+ T cells in the presence of adenosine (55). Moreover, the adenosine-A2AR signaling regulates PKA and mTORC1 activation and impairs the metabolic fitness of CD8+ T cells (56).

#### 2.2.2 NK Cells Respond to the Adenosine - A2AR Signaling

Natural killer (NK) cells, which can destroy target cells without antigen pre sensitization, have no MHC restriction and fast response speed in tumor immunity. Adenosine-mediated A2AR signal is an internal negative regulator of NK cell maturation and antitumor immune response. The adenosine- A2AR signaling limits NK cell maturation and proliferation. NK cell-specific A2AR-deletion, which promotes the maturation and proliferation of NK cells (7). The adenosine- A2AR signaling not only reduces the expression of proinflammatory factors (such as IFN-γ, IL-2, TNF-α, GM-GSF, and MIP-1α), it can also reduce the expression of perforin, which is essential for NC cells to play a cytotoxic role (57, 58). In addition, the cleavage ability of NK cells dependent on the FASL pathway was also significantly reduced during this process (59).

#### 2.2.3 Macrophages Respond to the Adenosine - A2AR Signaling

It is generally believed that macrophages are derived from monocytes. And macrophages will differentiate into M1 cells, mainly playing roles in promoting inflammation, sterilization, and anti-tumor, stimulated by factors such as TLR, TNF-α, IFN-γ, CSF2. Stimulated by cytokines like IL4, IL-10, IL-13, TGF-β, and PGE2, macrophages will differentiate into M2 subtype, which could inhibit inflammation and promote wound repair, but also promote tumor proliferation and metastasis (60). Macrophages polarize into an M2 phenotype due to increased IL-10 and decreased TLR, TNF-α, IFN-γ, which is caused by the activation of the adenosine-A2AR pathway (61). Macrophages are the key mediators of ADCP (antibody-mediated cellular phagocytosis) and can be observed in a large number in the tumor microenvironment (62, 63). Adenosine signaling can damage macrophage antibody-mediated ADCP, acting as a “don’t eat me signal”. In a xenograft lymphoma model, A2AR antagonist drug inhibition overcomes adenosine-mediated negative regulation of ADCP (64). In conclusion, the adenosine-A2AR signaling leads to the low pro-inflammatory and defensive immune activity of macrophages under tumor conditions, showing more phenotypic and functional characteristics of M2 type, and abnormally secreting growth factors such as VEGF. All these promote the proliferation and metastasis of tumors.

## 3 A2AR Antagonist and Clinical Progress

The discovery of the important role of the adenosine-A2AR pathway in the human body [not only in cancer but also in other diseases such as Parkinson’s disease (67, 68)], has made the development of effective and selective A2A adenosine receptor antagonists an appealing research field. Adenosine receptor antagonists can be divided into two groups: xanthine derivatives and nitrogen poliheterocyclic systems (69). However, some of these compounds have issues that prevent them from being used in clinical trials, such as low water solubility (SCH58261, and xanthine derivatives) or high affinity for the A2B adenosine receptor subtype (ZM241385) (69).

Currently, clinically tested A2AR antagonists include CPI-444, NIR178, AZD4635, etc (**Table 1**). The first clinical report demonstrating antagonistic effects of the adenosine pathway in cancer immunotherapy was published in 2020. In this clinical trial, 68 renal cell cancer patients (most of them resistant or refractory to anti-PD-1/PD-L1 antibodies and had predominantly PD-L1-negative tumors) received either ciforadenant or ciforadenant plus atezolizumab therapy. This study demonstrated the antitumor activity of both monotherapy and anti-PD-L1 combination therapy in refractory renal cell cancer patients. Median progression-free survival 4.1 months with monotherapy *vs* 5.8 months with combination and overall survival >69% at 16 months *vs >*90% at 25 months were observed. In many other trials, A2AR antagonists immunotherapy also has shown activity in many types of cancer patients (31, 33, 35). These encouraging findings support the adenosine axis as a feasible immunotherapy target, but further research is needed to establish which drug or combination would be most effective.

**Table 1:** A2AR selective antagonists in clinical trials for cancer Immunotherapy.

Drugs	Description	ClinicalTrials.gov identifier	Phase
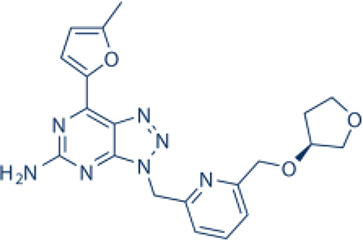 Ciforadenant (CPI-444)	A selective Adenosine A2A receptor antagonist that binds to A2AR with a K_i_ value of 3.54 nM and is more than 50 times more selective for A2AR than other Adenosine receptor subtypes (65).	NCT02655822 NCT04280328 NCT03337698 NCT03454451	1 1 1 1
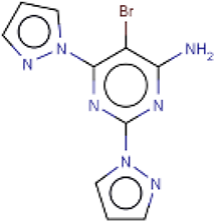 Taminadenant (NIR178)	A2AR antagonist	NCT03207867 NCT04895748 NCT03549000	2 1 1
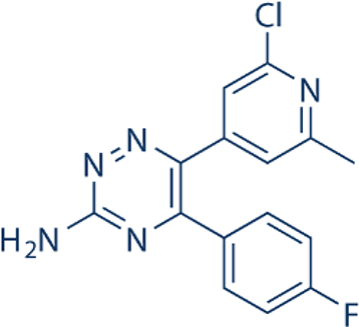 Imaradenant (AZD4635)	An oral A2AR antagonist that can bind to human A2AR with a K_i_ value of 1.7 nM. The selectivity for A2AR is more than 30 times that for other adenosine receptors (66)	NCT03381274 NCT04089553	1/2 2
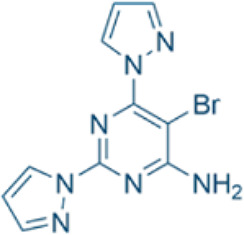 PBF-509	A non-xanthine potent and selective competitive antagonist of the human A2AR	NCT02403193	1/2
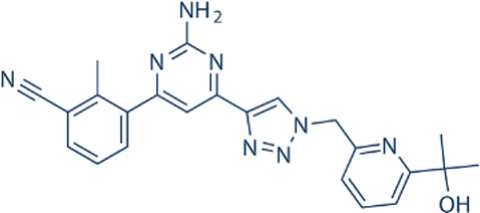 Etrumadenant (AB928)	A novel A2AR/A2BR dual active antagonist with K_d_ values of 1.4 nM and 2 nM for A2AR and A2BR, respectively (34)	NCT03720678 NCT03629756 NCT04262856 NCT04892875 NCT03719326 NCT04381832 NCT03846310 NCT04660812	1 1 2 1 1 1/2 1 1/2
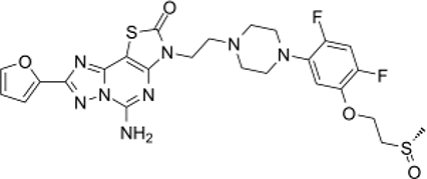 Inupadenant (EOS-850)	An oral, highly selective A2A receptor antagonist. Inupadenant cannot cross the blood-brain barrier (32)	NCT05060432	1/2
CS3005	A2AR antagonist	NCT04233060	1
EOS100850	A2AR antagonist	NCT03873883	1

**Figure 3:**
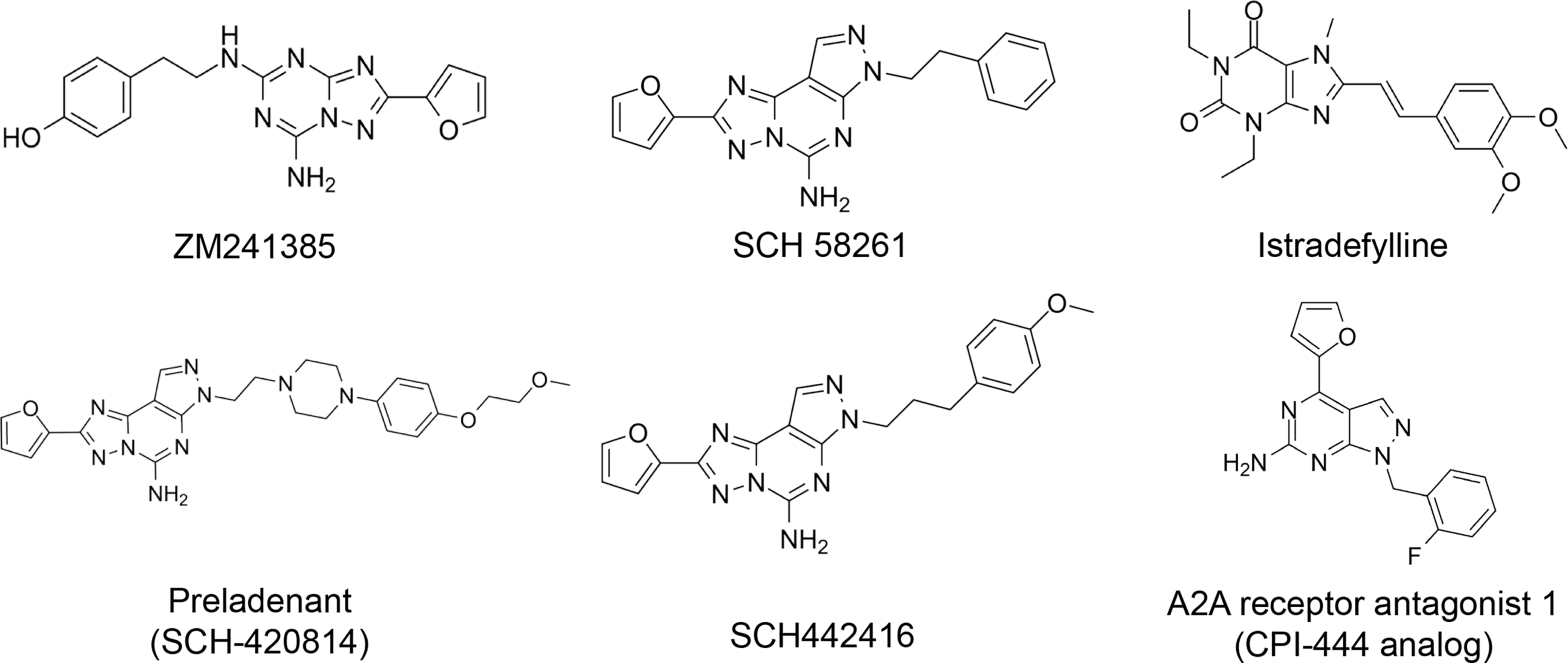
High affinity and selective adenosine A2AR antagonist.

Here, we list some other high affinity and selective adenosine A2AR inhibitors with potential clinical application in **Figure 3**. With a K_i_ value of 1.4 nM, ZM241385 is a strong, high affinity, and specific adenosine A2AR antagonist (70, 71). Istradefylline, with a K_i_ of 2.2 nM, is an extremely strong, selective, and orally active adenosine A2A receptor antagonist (72).. SCH58261 is a potent, selective, and competitive antagonist of adenosine A2A receptor with an IC50 of 15 nM, and displays 323-, 53- and 100-fold more selective for A2A receptor than A1, A2B, and A3 receptors, respectively (73–75). With a K_i_ of 1.1 nM and over 1000-fold selectivity over other adenosine receptors, Preladenant (SCH-420814) is a powerful and competitive antagonist of the human adenosine A2A receptor (76). With a K_i_ of 0.048 for human A2AR, SCH442416 is a strong, selective, and brain-penetrant antagonist of A2AR. It is 23145-, 208333-, and 208333-fold more selective for A2A receptor than A1, A2B, and A3 (77). With K_i_ values of 4 nM, A2A receptor antagonist 1 (CPI-444 analog) is a selective antagonist of adenosine A2A receptor and has over 66-fold selectivity over adenosine A1 receptor (78).

## Conclusion

Hypoxia-induced adenosine accumulation in the tumor microenvironment is common in a variety of tumors. The activation of the adenosine A2AR signaling pathway leads to immunosuppression and tumor EMT and anti-apoptosis, which promote the process of tumor development. A2AR antagonist blocking, or A2AR knockout of immune cells, indicates that blocking the adenosine-A2AR signaling pathway can significantly improve the antitumor effect. At present, A2AR inhibitors have shown gratifying effects in many clinical trials of cancer. However, for some patients, the lack of immune cells, or the mutation or deletion of cancer cell antigen leads to the unrecognition of T cell surface receptor TCR. As a result, A2AR inhibitors cannot exert their effect. The combination of A2AR inhibitors and CAR T therapy or other drugs can effectively improve the antitumor effect and greatly expand the application scenario of A2AR antagonists. Future combinations of A2AR inhibitors and CAR T may become the most effective and highly regarded cancer therapies.

## Author Contributions

All authors listed have made a substantial, direct, and intellectual contribution to the work and approved it for publication.

## Funding

This work was funded by the National Natural Science Foundation of China (NO. 11972099).

## Conflict of Interest

The authors declare that the research was conducted in the absence of any commercial or financial relationships that could be construed as a potential conflict of interest.

## Publisher’s Note

All claims expressed in this article are solely those of the authors and do not necessarily represent those of their affiliated organizations, or those of the publisher, the editors and the reviewers. Any product that may be evaluated in this article, or claim that may be made by its manufacturer, is not guaranteed or endorsed by the publisher.
